# Comparative Analysis of Stress Induced Gene Expression in *Caenorhabditis elegans* following Exposure to Environmental and Lab Reconstituted Complex Metal Mixture

**DOI:** 10.1371/journal.pone.0132896

**Published:** 2015-07-13

**Authors:** Ranjeet Kumar, Ajay Pradhan, Faisal Ahmad Khan, Pia Lindström, Daniel Ragnvaldsson, Per Ivarsson, Per-Erik Olsson, Jana Jass

**Affiliations:** 1 School of Science and Technology, The Life Science Center-Biology, Örebro University, Örebro, Sweden; 2 Boliden Mineral AB, Boliden, Sweden; 3 Envix Nord AB, Umeå, Sweden; 4 ALS Scandinavia AB, Täby, Sweden; CSIR-Central Drug Research Institute, INDIA

## Abstract

Metals are essential for many physiological processes and are ubiquitously present in the environment. However, high metal concentrations can be harmful to organisms and lead to physiological stress and diseases. The accumulation of transition metals in the environment due to either natural processes or anthropogenic activities such as mining results in the contamination of water and soil environments. The present study used *Caenorhabditis elegans* to evaluate gene expression as an indicator of physiological response, following exposure to water collected from three different locations downstream of a Swedish mining site and a lab reconstituted metal mixture. Our results indicated that the reconstituted metal mixture exerted a direct stress response in *C*. *elegans* whereas the environmental waters elicited either a diminished or abrogated response. This suggests that it is not sufficient to use the biological effects observed from laboratory mixtures to extrapolate the effects observed in complex aquatic environments and apply this to risk assessment and intervention.

## Introduction

Mining and mining processes contribute to the emission of transition metals into the environment. Transition metals including iron (Fe), zinc (Zn), copper (Cu), cobalt (Co), manganese (Mn) and molybdenum (Mo) are crucial micronutrients governing major biological processes such as the transport of biomolecules and epigenetic regulation of gene expression [1]. However, at concentration that exceed the physiological threshold, these metals may cause disease. In addition, toxic transition metals, such as cadmium (Cd), lead (Pb), arsenic (As) and mercury (Hg), accumulate in the body that leads to neurological and physiological dysfunction and disease [2]. Toxicological studies of transition metal exposure have effectively identified adverse effects on humans and animals, yet these substances continue to be released into the environment [3]. The speciation and diversity of transition metals present in the environment varies in a site-specific manner, calling for more targeted risk assessment and intervention. This has been recognized in the European water policy and Water Framework Directive (WFD 2000/60 EC) when establishing the bioavailability based environmental quality standards (EQS), thus stressing the need for site-specific considerations. The European Union’s new guidelines encourage incorporating bioavailability data in risk assessment and propose a tiered approach. However, when exceeding generic and local site specific EQS, other tools are needed to perform risk assessment of local contamination situations.

*Caenorhabditis elegans*, a free living bacterivore nematode residing in the interstitial liquid phase of soils with decomposing organic matter, has been used as an indicator organism of environmental toxicity [4]. It has been characterized genetically, physiologically and developmentally and many of its basic physiological and stress responses are conserved with higher organisms [5]. It is therefore a valuable predictive toxicological model. As approximately 60–80% of the *C*. *elegans* genes are homologous to those in humans, it can be used as a platform for analysis of environmental stress responses of importance to human diseases [4, 6]. This together with the easy maintenance, fully sequenced genome and the possibility to study physiological responses in relation to functional effects on gene expression makes *C*. *elegans* an attractive animal model for studying ecotoxicological disturbances.

*C*. *elegans* has been used for toxicity analysis of soil, water, sewage and effluents containing either organic substances or metals [7–10]. Most studies have focused on acute toxicity with lethality as an endpoint, often at high exposure concentrations that are normally not encountered in the environment [11,12]. More sensitive endpoints such as reproduction, growth, longevity, movement and locomotion are suitable for evaluating realistic exposure levels to toxic substances [13–16]. However, these require extended exposure times and may not detect subtle differences, therefore it is essential to develop techniques with more sensitive endpoint evaluation.

The present study used *C*. *elegans* as a model organism to compare the effect of metal toxicity in environmental samples with a laboratory reconstituted metal mixture on the expression of selected stress response genes. *C*. *elegans* were exposed to a metal cocktail (MC) of Zn, Cu and Cd at levels observed in Lake Hornträsket and environmental water collected from Lake Hornträsket and its tributary downstream of the Hornträsket mine in northern Sweden. These sites were selected for their naturaly decreasing environmental gradient of transition metals in the water system. The gradient started at Lake Hornträsket, a contaminated site near a mine. From Lake Hornträsket the water flowed through Vormbäcken stream and finally reached river Vindelälven, a Natura 2000 protected area (Council Directive 92/43/EEC) identified by the European Union. The biological effects of these exposures were evaluated on *C*. *elegans* using expression analysis of multiple genes previously reported in various organisms to have been affected by toxic substances. The genes in this study were selected from those associated with oxidative stress, metal toxicity, hormone regulation, development, apoptosis, antimicrobial response and immunity, largely involved in response to environmental toxicity. A qRT-PCR analysis of a limited number of genes was the chosen strategy due to increased sensitivity to traditional methods and a manageable data size in comparison to microarray studies.

## Materials and Methods

### Chemicals

The metal salts used in the study, namely NaCl, KCl, ZnCl_2_, CdCl_2_ and CuCl_2_, were obtained from Sigma Aldrich, USA. The salt solutions for the MC were prepared with Milli-Q water (Merck Millipore, Stockholm) to give a final concentration of 400μg/l Zn, 30 μg/L Cu and 0.5 μg/l Cd in K-medium. K-medium (51 mM NaCl and 32 mM KCl) was prepared as previously reported with Milli-Q water [17].

### Sample Site Description

Lake Hornträsket and waters downstream were an ideal environmental water system to evaluate the biological effects of transition metal emission from a mine. Lake Hornträsket has a small inflow of surface water, while approximately 80% of the inflow originates from groundwater. The lake is 6.6 km^2^ with an estimated volume of 35.84 Mm^3^ and a catchment area of approximately 40 km^2^. The estimated turnover time is 2.6 years with an outflow to the creek Vormbäcken ending at the river Vindelälven. There are three closed mines adjacent to lake Hornträsket that can still impact water quality. Hornträsket mine has the highest release of metals into the lake, while the Rävlidmyran mine, southwest of the lake, has more limited effects. In addition there is a mine located northeast of the lake, Granlunda mine that has no impact on the lake. Hornträsket was initially an underground mine for copper and zinc ore and became an open pit in 1988. Upon reclamation of the mine in 1996, the remediation involved refilling the mine with mineralized waste rock covered by 0.3–1 m of till. The first remedial measures during the 1990s were unsatisfactory, with leakage of acid mine drainage to the lake causing severe impact on aquatic life. A series of improved remedial actions performed during 2005–2012 [18] have resulted in a significant improvement of Lake Hornträsket water quality as evidenced by the return of some aquatic life, e.g. fish and benthic organisms.

### Sampling Locations and Chemical Analysis

**Fig 1:**
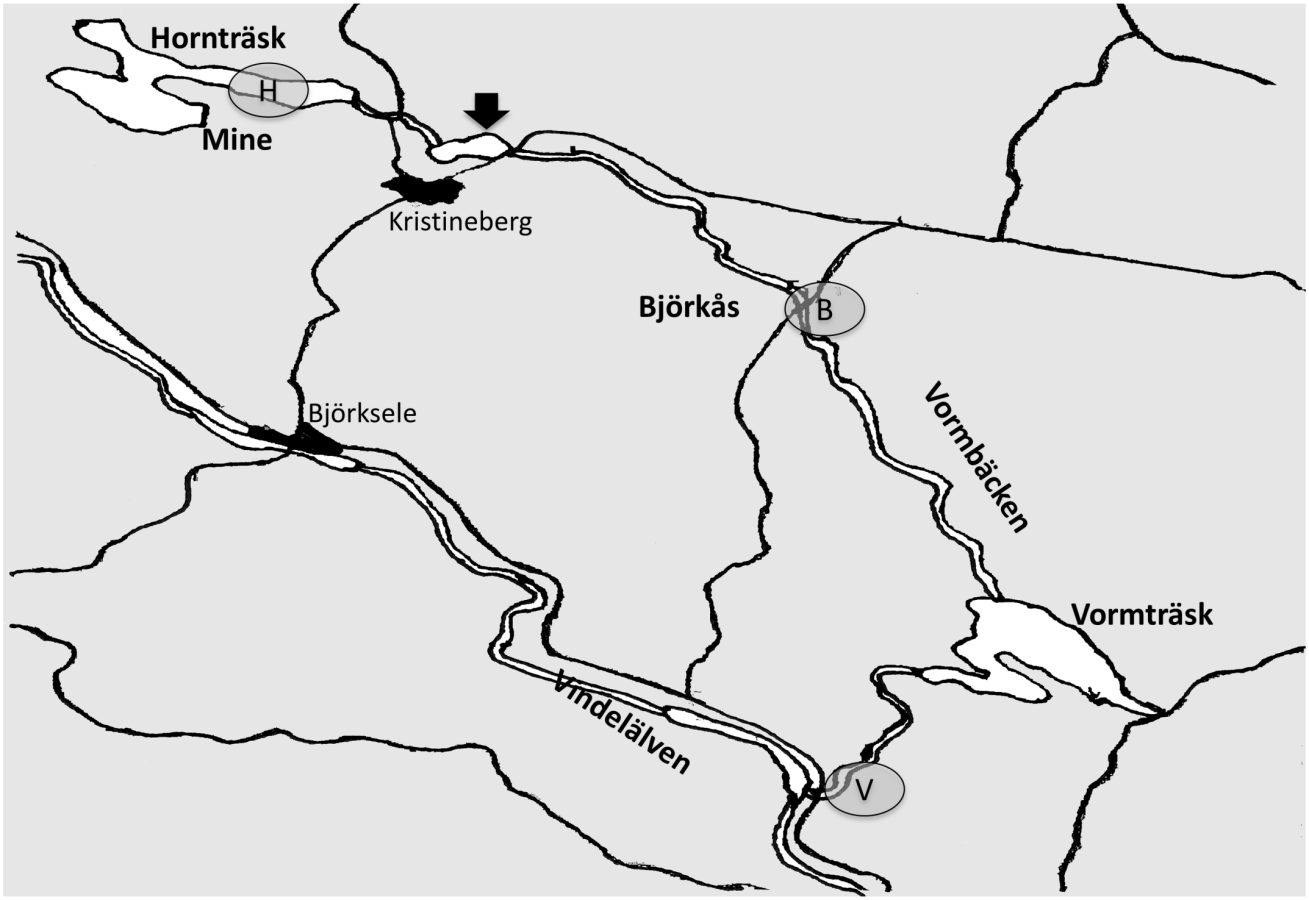
Map of lake Hornträsket and downstream Vormbäcken stream in northern Sweden. Water samples were collected from Lake Hornträsket (H) adjacent to Hornträsket mine (Mine) and downstream Vormbäcken stream at Björkås (B) and the exit to Vindelälven (V). The tailings dam is indicated by an arrow.

Waters were collected in sterile one liter bottles from three different location in the Hornträsket mining region in north Sweden. No permit or permission was required for the sampling of waters according to “the everyman’s right” (allemansrätten) in the Swedish constitution. Fig 1 shows the map of the sample locations; Lake Hornträsket east (site H; 65°05’29.19”N, 18°30’26.39”E) and Vormbäcken stream at Björkås (site B; 65°01’30.33”N, 18°44’12.25”E) and the exit into Vindelälven (site V; 65°52’43.27”N, 18°43’54.96”E). Adjacent to the southern part of Lake Hornträsket is the copper and zinc ore mine Hornträsket mine that was reclaimed in 1996. Metal inflow into the lake is primarily groundwater seepage from the closed mine. Water samples for chemical analysis were collected in bottles (glass for organic compounds and plastic for metals) and sent for analysis to ALS Scandinavia AB (Täby, Sweden, www.alsglobal.se). Organic compounds were analyzed by GC-MS while inorganic substances were analyzed by inductive coupled plasma (ICP)—atomic emission spectrometry, high resolution sector-field mass spectrometry (ICP-SFMS) and atomic fluorescence spectroscopy (AFS) according to accredited protocols.

### Preparation and Synchronization of Nematode Culture

The Bristol wild type N2 strain of *C*. *elegans* (kind gift from Dr. Peter Swoboda, Karolinska Institute) was maintained on nematode growth medium plates (NGM) seeded with a lawn of *Escherichia coli* OP50 at 20°C according to the standard protocol [19]. For gene expression studies, the *C*. *elegans* were age synchronized as follows. The nematodes were grown on multiple NGM plates (90 mm or 60 mm) at 20°C to obtain a large number of gravid adult hermaphrodites and harvested into 15 ml centrifuge tubes using M9 buffer. The worms were lysed with an alkaline-bleach mixture (final concentration 0.5 N NaOH, 1% HOCl) until most of the eggs were released from the worms, approximately 15 min. The eggs were recovered by centrifugation (2400x*g*) and washing twice with distilled water, followed by one wash with K-medium before being transferred to fresh NGM plates. The eggs were hatch overnight to obtain growth-arrested L1 larvae. The L1 worms were recovered in K-medium, transferred onto multiple *E*. *coli* seeded NGM plates and grown at 20°C for 48h until they reached L4/young adult stage. Synchronized L4/young adult worms were harvested from the plates in K-medium and used in the subsequent studies.

### Experimental Design and RNA Preparation

Age synchronized L4/young adult worms were washed in K-medium and exposed to K-medium prepared with Milli-Q water (control), water from the three environmental sites or the MC containing Zn, Cu and Cd concentrations measured in lake Hornträsket (site H; 400 μg/l Zn, 30 μg/l Cu and 0.5 μg/l Cd). A lethality assay was first performed using approximately 20 worms per well containing 2ml of K-medium prepared with Milli-Q water (control) or environmental waters in the absence of food and incubated at 20°C. The number of live/dead worms were counted after 24 h. For the exposure study, approximately 1000 worms were incubated for 6 h at 20°C in 2 ml of K-medium prepared with the Milli-Q water (control), the different environmental waters or the MC in the absence of food in a 12-well cell culture plate (BD Biosciences, USA). Four replicates for each condition were done. The treated worms were collected by centrifugation at 2400x*g*, homogenized in TRI reagent (Sigma Aldrich, USA) using Minilys (Bertin Technologies, USA) and the RNA was isolated as per the manufacturer’s protocol. The RNA was further treated with Heat and Run DNase (ArcticZymes, Norway) to remove DNA contamination and quantified using a NanoVue spectrophotometer (GE Healthcare, UK).

### cDNA Synthesis and qPCR

Complementary DNA (cDNA) was synthesized according to the qScript cDNA synthesis kit (Quanta Biosciences, USA) using 1 μg of RNA in a 20 μl reaction. The qPCR was performed on 1 μl cDNA (10 ng) using the KAPA SYBR Fast qPCR kit (Kapa biosystems, USA) and 0.1 pM of each primer in a 20 μl reaction. The reactions were prepared using CAS-1200 automated PCR Robot (Corbett life Sciences, UK) in 96 well format. The thermo-cycling conditions included, one cycle of denaturation at 95°C for 5 min, followed by 45 cycles of denaturing at 95°C for 2 sec and annealing/extension at 60°C for 30 sec. The Ct values were normalized against the Y45F10D.4 gene encoding for the putative iron-sulfur cluster assembly enzyme (*fes*) and the relative gene expression was determined using the ΔΔCt method [20, 21]. The reference gene was selected from preliminary studies evaluating the stability of 3 different reference genes Y45F10D.4, *rps-18* and *act-1* using GeneNorm (data not shown). The 46 genes used in the study are listed with their respective functions in Table 1.

**Table 1:** Target genes used in the qPCR analysis.

Gene code	Transcript ID	Protein product	Function
**Heat shock**			
*hsf-1*	Y53C10A.12.1	Heat shock factor 1	Transcription factor
*hsp-16*.*1*	T27E4.2	Heat shock protein 16.1	Heat shock, stress
*hsp-16*.*2*	Y46H3A.3	Heat shock protein 16.2	Heat shock, stress
*hsp-16*.*48*	T27E4.3	Heat shock protein 16.48	Heat shock, stress
*hsp-70*	C12C8.1	Heat shock protein 70	Heat shock, stress
*sip-1*	F43D9.4	Stress- induced protein 1	Embryonic lethality, Lifespan
**Oxidative stress**			
*gst-4*	K08F4.7	Glutathione-S-transferase 4	Oxidative stress
*sod-1*	C15F1.7b.1	Superoxide dismutase [Cu-Zn]	Oxidative stress
*cat-2*	Y54G11A.6	Catalase 2	Oxidative stress
*cyp-35A2*	C03G6.15	Cytochrome P450-35A2	Xenobiotic response
*daf-2*	Y55D5A.5b	Insulin like growth factor-1 receptor-2	Environmental stress
*nnt-1*	C15H9.1	Nicotinamide nucleotide transhydrogenase	Oxidative stress
*osr-1*	C32E12.3	Osmotic stress resistance protein	Oxidative stress
*skn-1*	T19E7.2a.1	Protein skinhead-1	Oxidative stress
*prdx-2*	F09E5.15a.1	Peroxiredoxin-2	Oxidative stress
*kel-8*	W02G9.2.2	Kelch-like protein 8	Oxidative stress
*gcs-1*	F37B12.2.2	Gamma-glutamylcysteine synthetase	Oxidative stress
**Metal response genes**			
*aip-1*	F58E10.4	Arsenite inducible protein	Arsenic toxicity, stress
*cdf-2*	T18D3.3	Cation diffusion factor-2	Zinc transport
*cdr-1*	F35E8.11	Cd-inducible lysosomal protein-1	Cadmium stress
*ftn-1*	C54F6.14	Ferritin heavy chain homolog1	Iron homeostasis
*hif-1*	F38A6.3e	Hypoxia-induced factor 1	Metal toxicity, antimicrobial
*hmt-1*	W09D6.6	Heavy metal tolerance factor 1	Metal toxicity
*mtl-1*	K11G9.6	Metallothionein-1	Metal toxicity
*mtl-2*	T08G5.10	Metallothionein-2	Metal toxicity
*numr-1*	F08F8.5	Nuclear localized Metal Responsive family member-1	Metal toxicity, lifespan
*pgp-5*	C05A9.1a	P-glycoprotein-5	Metal toxicity, antimicrobial defense
**Hormone receptor and development**			
*apl-1*	C42D8.8a	Beta-amyloid-like protein-1	Development
*daf-12*	F11A1.3c	Dauer formation-12	Hormone receptor
*fem-1*	F35D6.1b	Feminization of XX and XO animals -1	Sex determination
*fshr-1*	C50H2.1	Follicle stimulating hormone receptor-1	Hormone receptor
*nhr-14*	T01B10.4a.1	Nuclear hormone receptor-14	Hormone receptor
*nhr-8*	F33D4.1b	Nuclear hromone receptor-8	Hormone receptor
*vit-2*	C42D8.2a.1	Vitellogenin-2	Embryonic development
*vit-6*	K07H8.6a	Vitellogenin-6	Embryonic development
**Innate immunity and apoptosis**			
*abf-2*	C50F2.10	Antibacterial Factor related-2	Microbicidal activity
*ape-1*	F46F3.4d	apurinic/apyrimidinic endonuclease 1	Apoptosis
*bar1*	C54D1.6	Beta-catenin/armadillo-related protein 1	Wnt signaling
*cep-1*	F52B5.5a.1	Transcription factor cep-1	Apoptosis
*clec-60*	ZK666.6	C-type lectin-60	Antimicrobial defense
*lys-7*	C02A12.4.2	Lysozyme-7	Antimicrobial response
*tir-1*	F13B10.1d.1	Toll and Interleukin 1 Receptor-1	Innate immune response
*tol-1*	C07F11.1	Toll like receptor-1	Innate immunity
*wah-1*	Y56A3A.32	Worm apoptosis inducing factor-1	Apoptosis
**Reference gene**			
Y45F10D.4	Y45F10D.4	Fes, iron-sulfur cluster assembly enzyme	Embryonic and larval viability Reference gene

### Statistical Analysis and Biotic Ligand Model

Statistical analyses were performed using one way analysis of variance (ANOVA) followed by Tukey’s post-test for multiple group comparison with Graphpad Prism 5 software (Graphpad software, USA). Unless otherwise mentioned ‘*’ refers to *p*< 0.01 as compared to the control, whereas ‘#’ represents differences between groups. Data (n = 4) was plotted relative to the control levels and shown as mean ± standard deviation (SD). The multivariate data analysis and principal component analysis (PCA) were performed using the SIMCA software version 13.0.3 (Umetrics, Sweden) at a significance level of 0.05. A score plot showing sample grouping and a loading plot indicating the relationships between the variables was used in the analysis. Values that explain the variation, R^2^X values >0.7 and Q^2^ values >0.4 were considered to denote an acceptable model when analyzing biological data [22].

The toxicity of single metals in the environmental waters was modeled using the biotic ligand model (BLM) and available water chemical parameters including pH, hardness and dissolved organic carbon. The BLM was used to calculate the bioavailability of Cu and Zn in the present water samples using the program, Bio-met bioavailability tool version 2.3-04-12-2013 (http://bio-met.net/).

## Results

### Metal Contaminants in Environmental Waters

The chemical analysis of the water collected from the three locations in- and down-stream of Lake Hornträsket are presented in Table 2. The most significant source of metal emission into Lake Hornträsket is the reclaimed Hornträsket mine, creating a natural downstream gradient of toxic heavy metals. As anticipated, Zn, Cu, Pb, Ni and Cd concentrations were highest in Hornträsket lake nearest the mine and this decreased downstream at the other two sample sites. It was however interesting to note that the levels of all other metals measured were highest in Vombäcken stream at Björkås when compared to the other 2 sites, with Ca, Si, Mg, Na and K at mg/l concentrations. An increase in the concentrations of these metals was observed at the tailings dam which has its outflow into Vormbäcken between Hornträsket and Björkås. There were no major organic toxicants detected in the three environmental sites (below detection limits; Table 2 footnote).

**Table 2:** Chemical analysis of water samples from Lake Hornträsket and the Vormbäcken stream.

	Sample Sites (μg/l)		
Substance^a^	Hornträsket (H)	Vormbäcken at Björkås (B)	Vormbäcken exit at Vindelälven (V)
**pH**	7.76	7.10	7.29
**Ca**	6180 ± 740	39700 ± 4700	11400 ± 1400
**Si**	1780 ± 260	2700 ± 390	2420 ± 350
**Mg**	1750 ± 220	3910 ± 480	1290 ± 160
**Na**	1170 ± 240	2830 ± 420	1520 ± 270
**K**	762 ± 147	1660 ± 230	807 ± 149
**Zn**	407 ± 59	169 ± 24	36.9 ± 6.6
**Fe**	209 ± 28	990 ± 131	504 ± 67
**Al**	82.2 ± 13.7	98.3 ± 16.4	72.1 ± 12.4
**Mn**	55.4 ± 6.7	80.2 ± 9.7	58.0 ± 7.0
**Cu**	29.3 ± 5.0	15.4 ± 2.6	6.13 ± 1.04
**Sr**	16.2 ± 2.0	50.5 ± 6.3	20.5 ± 2.5
**Ba**	7.96 ± 1.01	9.80 ± 1.23	7.46 ± 0.97
**P**	4.43 ± 1.01	4.30 ± 0.93	4.20 ± 0.94
**Pb**	3.56 ± 0.57	0.762 ± 0.123	0.182 ± 0.031
**Ni**	1.78 ± 0.31	1.05 ± 0.19	0.541 ± 0.128
**As**	0.897 ± 0.164	3.80 ± 0.65	1.65 ± 0.29
**Cd**	0.497 ± 0.067	0.341 ± 0.045	0.0675 ± 0.0102
**Co**	0.266 ± 0.049	0.366 ± 0.068	0.0989 ± 0.0235
**Cr**	0.217 ± 0.043	0.323 ± 0.061	0.243 ± 0.047
**Mo**	0.0780 ± 0.0390	0.306 ± 0.065	0.206 ± 0.051
**Hg**	0.0036 ± 0.0008	0.0077 ± 0.0010	0.0031 ± 0.0007

^a^The following substances were below detection limits: aliphatics >C10-C12, >C12-C16, >C16-C35; chlorobenzine; total PCB; naphthalene; acenaphthylen; acenaphthene; fluorene; phenanthrene; anthracene; fluoranthene; pyrene; benzo(a)anthracene; chrysene; benzo(b)fluoranthene; benzo(k)fluoranthene; benzo(a)pyrene; benzo(ah)anthracene; benzo(ghi)perylene; indeno(123cd)pyrene; total PAH.

### Reconstituted Metal Cocktail and Environmental Water Samples Were Not Lethal to *C*. *elegans*

To evaluate the differential gene expression of *C*. *elegans* exposed to the MC and waters from Lake Hornträsket and Vormbäcken stream at Björkås and at the outflow into Vindelälven, we first determined if the waters induced acute toxicity in *C*. *elegans*. The MC contained 400 μg/l Zn (6.12 μM), 30 μg/l Cu (0.47 μM) and 0.5 μg/l Cd (4.5 nM), reconstituted according to the levels measured in Hornträsket lake, the highest level measured of the original environmental water samples. Synchronized L4/young adult worms exposed to either the environmental waters or MC for up to 24 h showed no lethality. In order to avoid bacterial interactions with the metals in solution, the *C*. *elegans* were exposed in the absence of food, however long exposure time may lead to a starvation response that could mask the metal response, therefore we limited the exposure to 6 h. This also allowed us to evaluate the early response genes in *C*. *elegans* and avoid variations in gene expression due to different developmental stages for qRT-PCR analysis.

### Induction of Heat Shock Response Genes by Metal Solution

**Fig 2:**
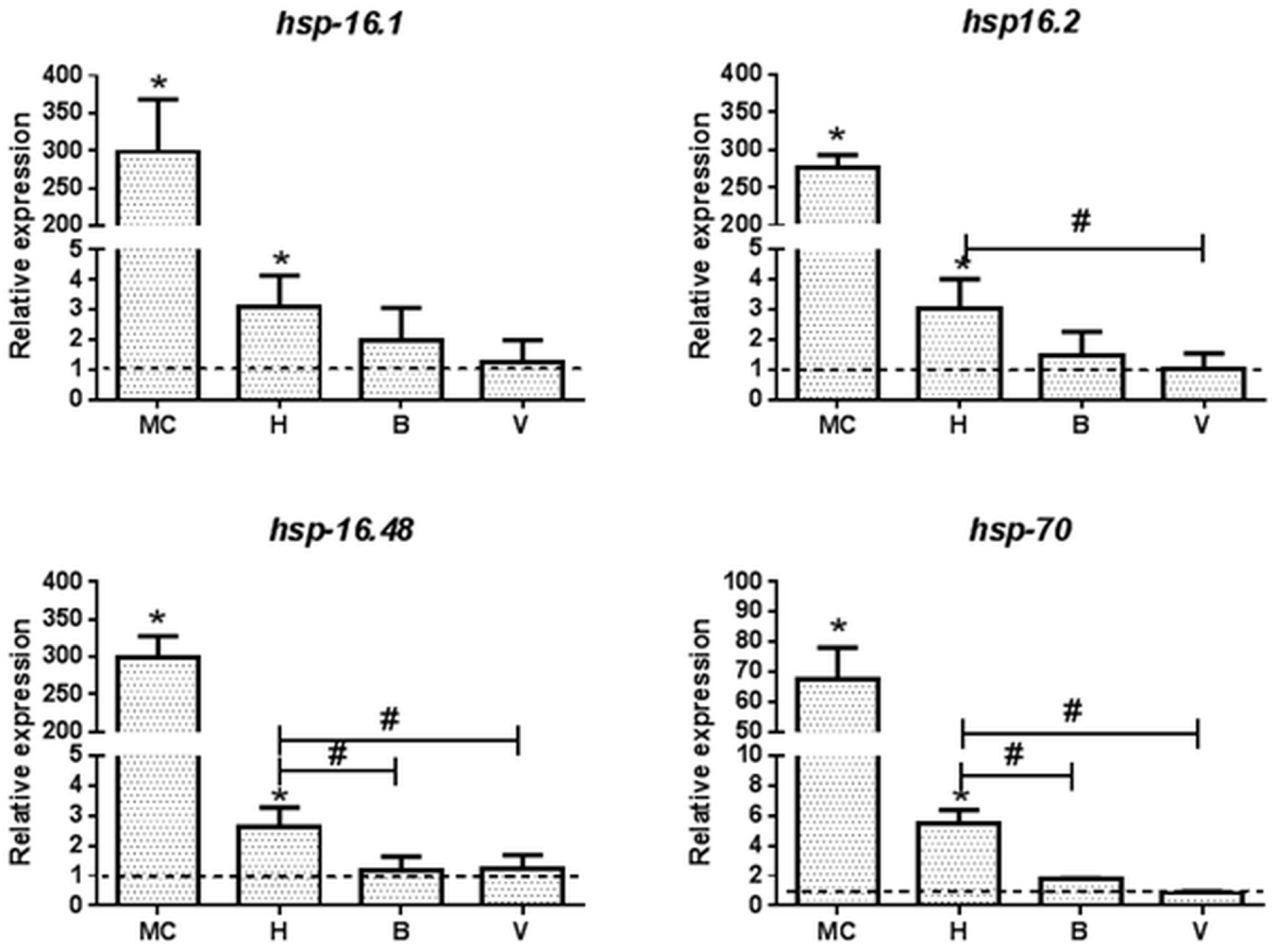
Metal exposure alters the expression of heat shock response genes in *C*. *elegans*. Synchronized young adult *C*. *elegans* were exposed for six hours to the metal cocktail (MC) or waters from Lake Hornträsket (H) and Vormbäcken stream at Björkås (B) and the exit to Vindelälven (V). qRT-PCR was performed for genes *hsp-16*.*1*, *hsp-16*.*2*, *hsp-16*.*48* and *hsp-70*. One way analysis of variance (ANOVA) followed by Tukey’s post-test was used for multiple group comparison where * refers to *p*<0.01 as compared to control, and # represents significant differences between the environmental sites H, B and V. The dotted line represents the expression level normalized to that of the control (K-medium prepared with Milli-Q water).

The up-regulation of heat shock proteins is a key response to general environmental stress that can be induced by exposure to toxic substances such as metals. Many of the heat shock proteins are chaperones involved in protein folding and protein stabilization. In this study, four genes for heat shock proteins and one general stress response transcription factor were included in the analysis as key representatives of this group. Significant up-regulation of four of the heat shock response genes was observed in *C*. *elegans* treated with the MC water. Genes *hsp*-*16*.*1*, *hsp-16*.*2* and *hsp-16*.*48* increased more than 250 fold, whereas *hsp-70* increased 65-fold upon exposure to the MC (Fig 2). Lake Hornträsket water, containing the same levels of Zn, Cu and Cd had a significant but much lower induction of the heat shock response genes. Two other genes, *sip-1* and *hsf-1*, encoding for a small heat shock protein and a transcription factor, respectively, did not show any significant changes as compared to the control (S1 Fig). Vormbäcken stream at Björkås and exit to Vindeläven did not induce any of the heat shock response genes above background level (Fig 2). Thus the stress response of the *C*. *elegans* exposed to the MC and the Lake Hornträsket water showed a similar trend, albeit Lake Hornträsket induced a much lower level even though the metal concentrations of Zn, Cu and Cd were equal.

### Oxidative Stress Response Genes Affected upon Exposure to Metal Mixtures

**Fig 3:**
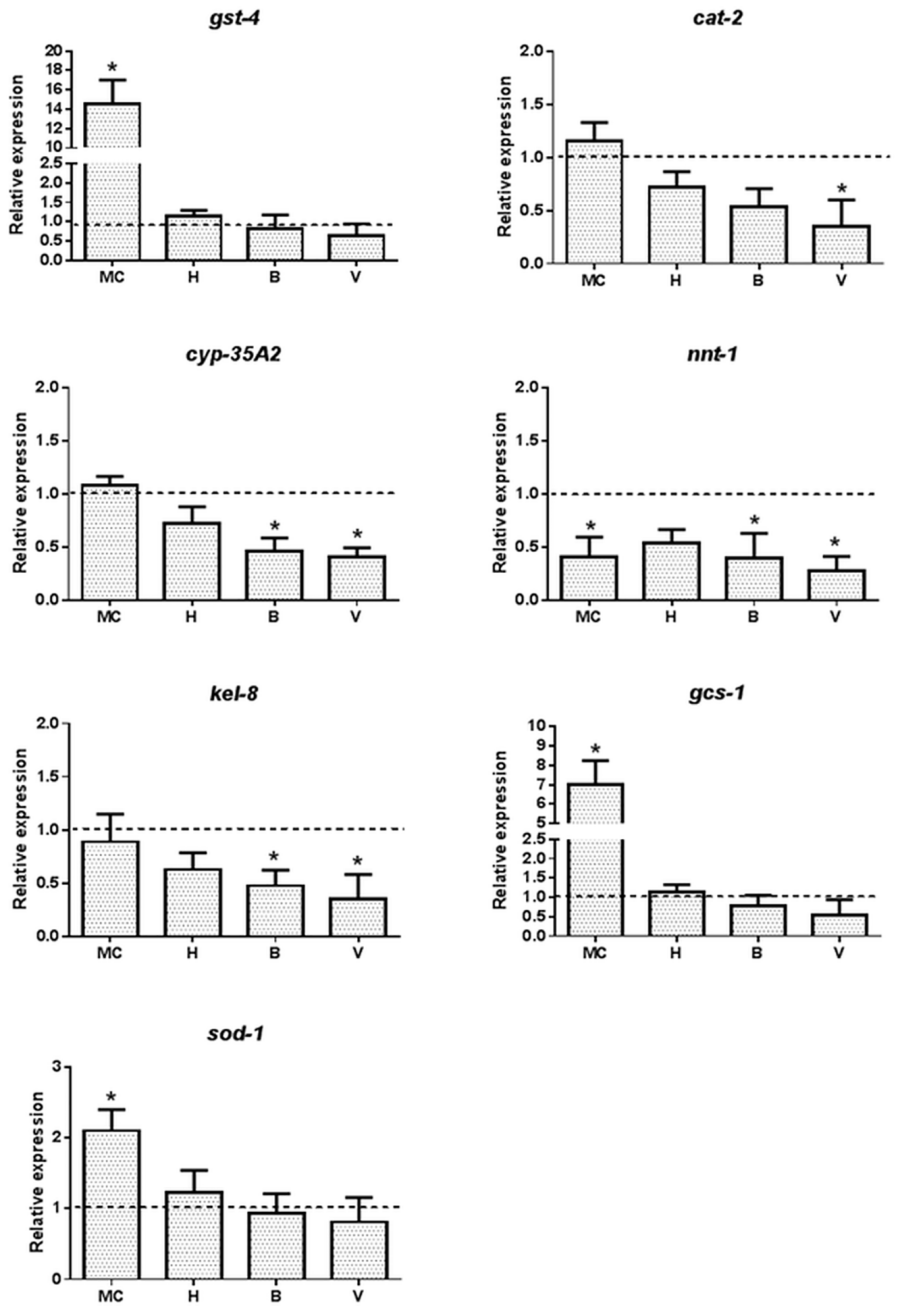
Exposure of *C*. *elegans* to metals affects the expression of oxidative stress response genes. Synchronized *C*. *elegans* treated with the metal cocktail (MC) or waters from Lake Hornträsket (H) and Vormbäcken stream at Björkås (B) and the exit to Vindelälven (V) were analyzed for the expression of *gst-4*, *sod-1*, *cat-2*, *cyp-35A2*, *daf-2*, *nnt-1*, *kel-8* and *gcs-1* using qRT-PCR. One way analysis of variance (ANOVA) followed by Tukey’s post-test was used for multiple group comparison where * refers to *p*<0.01 as compared to control and # represents significant differences in between sites H, B and V. The dotted line represents the expression level normalized to that of the control (K-medium prepared with Milli-Q water).

A mechanism of metal-induced toxicity is the generation of reactive oxygen species, which in turn activates the oxidative stress response in an organism [23]. Accordingly, 11 genes associated with oxidative stress and detoxification were included in the analysis. There were significant changes in expression in 7 of the 11 genes associated with oxidative stress response after exposure to the MC or the environmental waters (Fig 3). *C*. *elegans* treated with the MC had significant induction of *gst-4* (15-fold), *gcs-1* (6 fold) and *sod-1* (2-fold), while no significant changes in expression were observed after treatment with the environmental waters. The gene *gst-4* encodes for glutathione-S-transferase, a crucial modulator of oxidative stress response, *gcs-1* encodes for a rate limiting enzyme in glutathione metabolism called gamma-glutamine cysteine synthetase and *sod-1* encodes for superoxide dismutase, all involved in resistance to oxidative stress. In addition, *nnt-1*, which encodes for nicotinamide nucleotide transhydrogenase was down regulated by MC, while no significant changes were observed for the other genes. Hornträsket lake water (H) contained the same metal concentration as the MC, yet did not present a significant change in expression of any of the genes associated with oxidative stress. Additionally, significant down regulation of *cyp-35A2*, *nnt-1* and *kel-8* was observed after treatment with waters from both sites in Vormbäcken stream at Björkås (B) and exit to Vindelälven (V), while catalase *cat-2* was down regulated only by water from the exit to Vindelälven. The MC and site B and H had reduced levels of *nnt-1* expression. Genes *osr-1*, *skn-1* and *prdx-2* did not show any significant changes in expression from background levels (S1 Fig).

### Exposure Influenced the Metal Response Gene Expression

**Fig 4:**
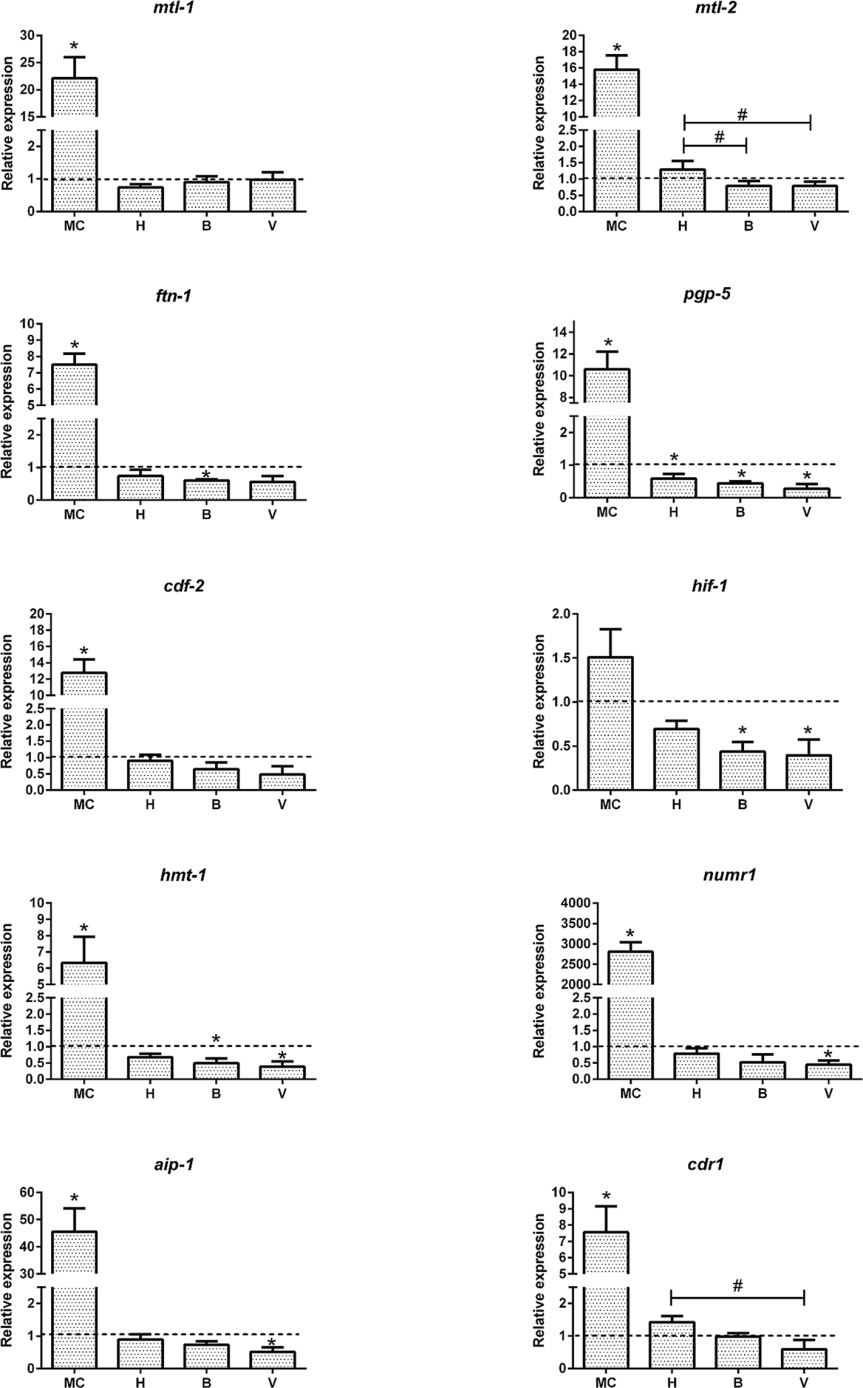
Effect of *C*. *elegans* exposure on the metal response genes. Adult *C*. *elegans* treated with the metal cocktail (MC) or waters from Lake Hornträsket (H) and Vombäcken stream at Björnås (B) and the exit to Vindelälven (V) were analyzed for the expression of ten metal response genes; *mtl-1*, *mtl-2*, *ftn-1*, *pgp-5*, *cdf-2*, *hif-1*, *numr-1*, *aip-1*, *cdr-1*, and *hmt-1*. One way analysis of variance (ANOVA) followed by Tukey’s post-test was used for multiple group comparison where * refers to *p*< 0.01 as compared to control and # represents significant differences in between the sites H, B and V. The dotted line represents the expression level normalized to that of the control (K-medium prepared with Milli-Q water).

The metal responsive genes group includes those directly influenced by metals and demonstrate that the organism senses toxic levels of metals in their environment. Genes previously shown to respond to heavy metals in different organisms were included in this study to compare the laboratory MC and environmental waters [1]. Most of the metal responsive genes were significantly up regulated in *C*. *elegans* after exposure to the MC (Fig 4). Metallothioneins encoded by *mtl-1* and *mtl-2* in *C*. *elegans* were induced 22- and 15-fold, respectively. Metallothionein genes are up regulated by Zn and Cu in order to maintain metal homeostasis, but also respond to other heavy metals as a toxic response by acting as scavengers for reactive oxygen species [24]. *ftn-1* encodes for ferritin, a protein responsible for iron homeostasis and iron toxicity was induced 6-fold, while hypoxia inducing factor *hif-1*, which is negatively regulated by *ftn-1*, was not altered by the MC. The genes *pgp-5*, *cdf-2*, *hmt-1* and *cdr-1* were collectively up regulated 5- to 15-fold by the MC. The nuclear localized metal responsive gene *numr-1*, which is a transcription factor, was up regulated more than 3000-fold and the arsenic response gene *aip-1* was induced more than 40-fold by the MC.

In contrast to the MC, exposure to the environmental waters resulted in either a very low reduction or no change in the examined genes (Fig 4). While *pgp-5* was up regulated 10-fold by MC, exposure to Lake Hornträsket water at the same metal concentrations lead to a small but significant down regulation of *pgp-5*. The metallothionein genes, *mtl-1* and *mtl-2* did not show any significant change compared to the control following exposure to the environmental waters. However, the *mtl-2* levels were significantly higher in Lake Hornträsket (H) than in Vormbäcken at Björkås (B) or at the outflow to Vindelälven (V). The two stream waters (B and V) down regulated the hypoxia inducing factor *hif-1* and the transition metal tolerance factor *hmt-1*. Thus environmental waters did not produce the same gene responses as the MC, even at the same metal concentration.

### Regulation of Nuclear Hormone Receptors and Developmental Genes

**Fig 5:**
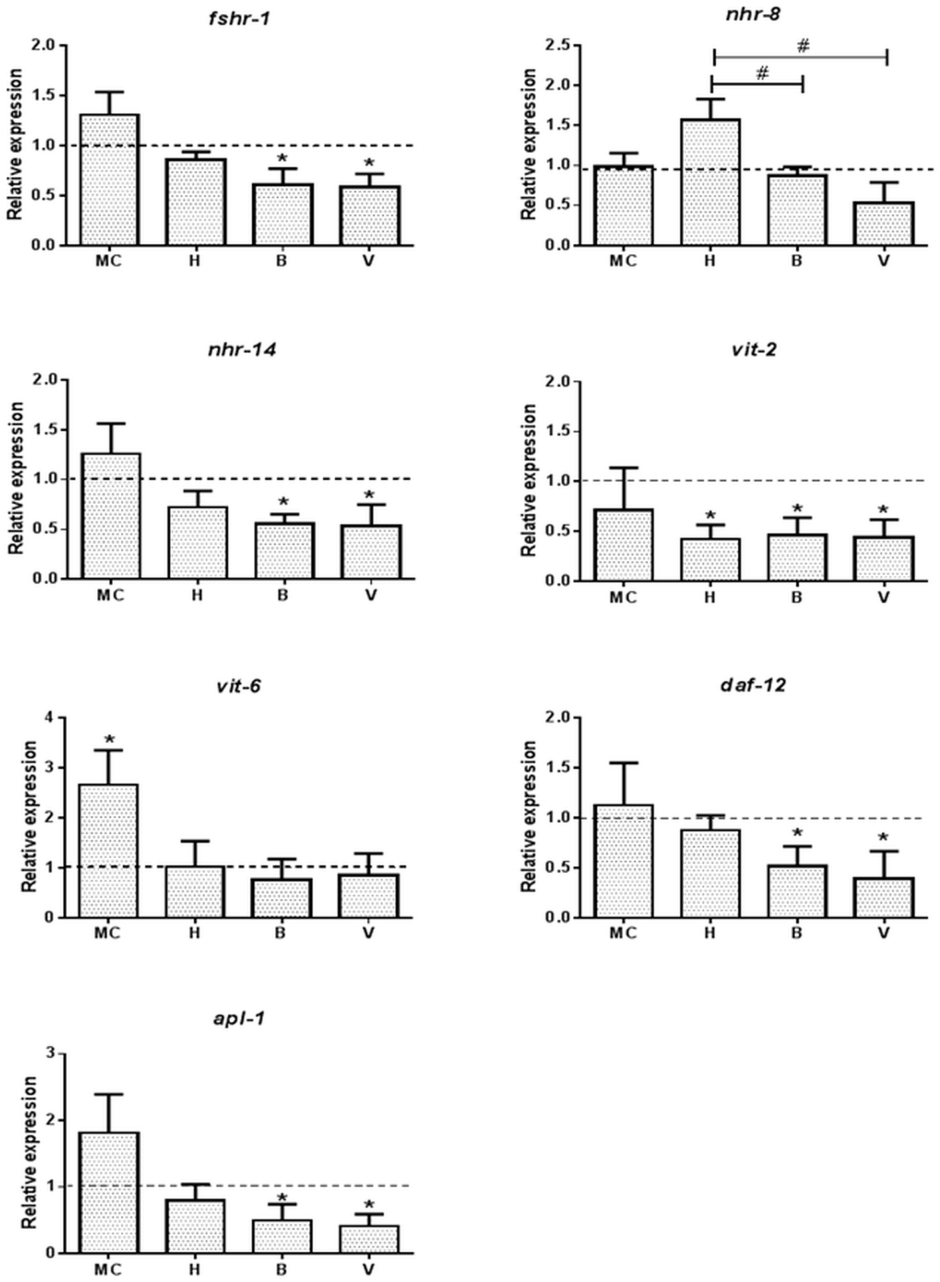
Effect of metals exposure on nuclear hormone receptors and developmental genes in *C*. *elegans*. *C*. *elegans* treated with the metal cocktail (MC) or waters from Lake Hornträsket (H) and Vombäcken stream at Björnås (B) and the exit to Vindelälven (V) were analyzed for the expression of genes associated with hormone receptors (*fshr-1*, *nhr-8* and *nhr-14)* and development (*vit-2*, *daf-12*, and *apl-1)* were tested using qRT-PCR. One way analysis of variance (ANOVA) followed by Tukey’s post-test was used for multiple group comparison where * refers to *p*<0.01 as compared to control whereas # represents significant differences in between the sites H, B and V. The dotted line represents the expression level normalized to that of the control (K-medium prepared with Milli-Q water).

Metals affect reproduction and development in *C*. *elegans*, therefore we included 8 genes encoding for nuclear hormone receptors and embryonic development [15]. The nuclear hormone receptors *fshr-1* and *nhr-14* were down regulated by both waters from Vormbäcken (B and V), however, none of the nuclear hormone receptors (*fshr-1*, *nhr-8* and *nhr-14)* were affected by the MC (Fig 5). The MC significantly up regulated vitellogenin *vit6*, a gene associated with developmental, while it was unaffected by exposure to Lake Hornträsket and Vormbäcken waters (Fig 5 and S1 Fig). All of the environmental waters down regulated *vit-2* 2.5-fold, while only Vormbäcken (B and V) water down regulated the insulin-IGF receptor homolog *daf-12* and the amyloid precursor-like protein *apl-1* ~ 2- to 3-fold in the *C*. *elegans*.

### Altered Expression of the Key Modulators Involved in Apoptosis and Innate Immunity

**Fig 6:**
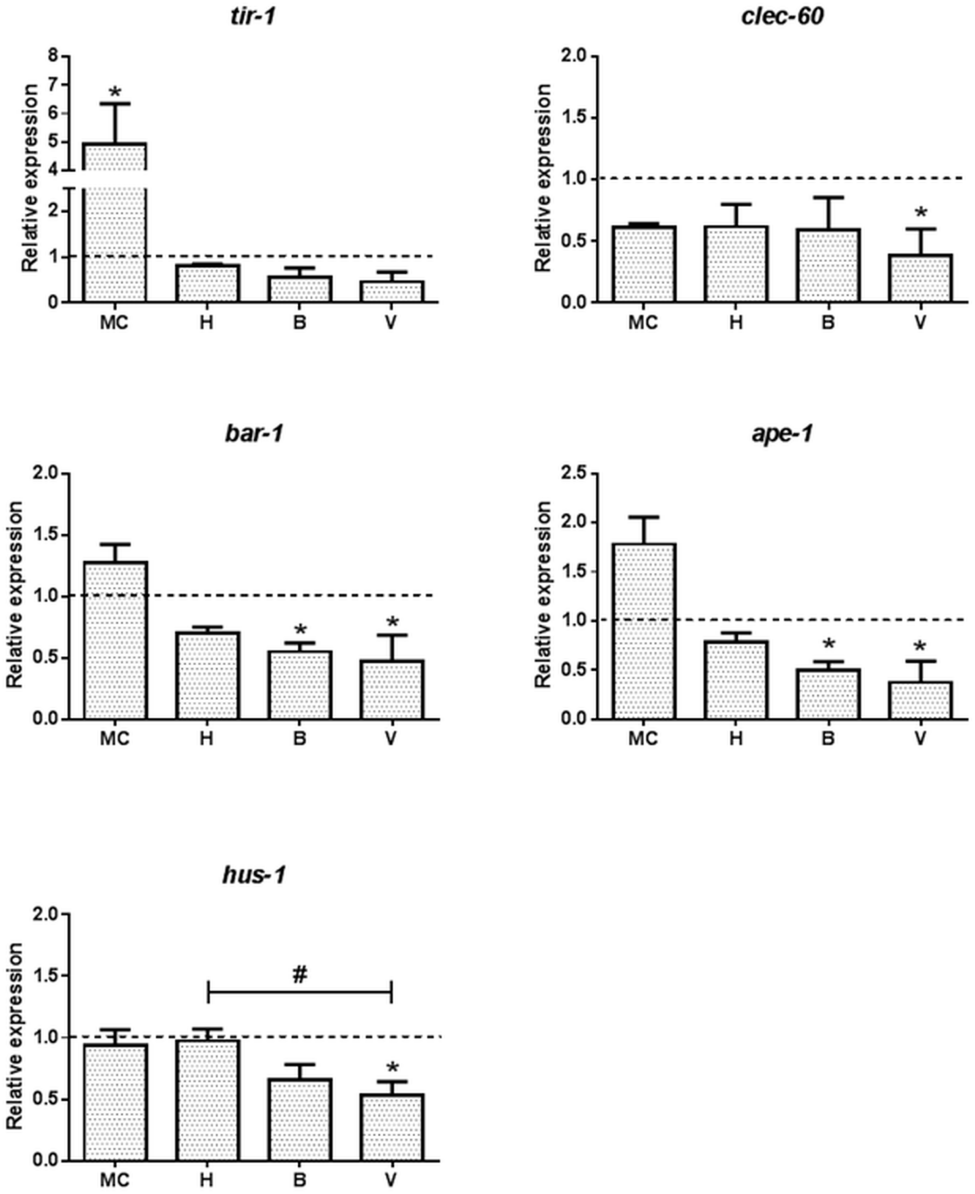
Effect of metal exposure on *C*. *elegans* genes involved in apoptosis and innate immunity. Apoptosis marker genes *ape-1* and *hus-1* and innate immunity modulators *tir-1*, *clec-60*, and *bar-1* were analysed in *C*. *elegans* treated with the metal cocktail (MC) or waters from Lake Hornträsket (H) and Vombäcken stream at Björnås (B) and the exit to Vindelälven (V). One way analysis of variance (ANOVA) followed by Tukey’s post-test was used for multiple group comparison where * refers to *p*<0.01 as compared to control and # represents significant differences in between the sites H, B and V. The dotted line represents the expression level normalized to that of the control (K-medium prepared with Milli-Q water).

Environmental stress is often involved in activating the innate immune response and apoptosis. Five genes associated with innate immunity and apoptosis were affected by exposure to either the MC or the environmental water samples, although none of them were conjointly affected by both exposures (Fig 6 and S1 Fig). Of the 6 genes involved in innate immunity (*tir-1*, *clec-60*, *bar-1*, *lys-7*, *abf-2* and *tol-1*), TIR motif-containing protein *tir-1* was significantly up regulated 5-fold in *C*. *elegans* treated with MC, but not by the environmental waters. The most downstream site, at the outflow to Vindelälven (V), down regulated *clec-60*, *bar1*, *ape-1* and *hus1*, while at Björkås, *bar-1* and *ape-1* were down regulated. Neither Lake Hornträsket water nor the MC exposure altered the expression of the genes associated with apoptosis (Fig 6 and S1 Fig). No change in expression was seen in *lys-7*, *abf-2*, *tol-1*, *cep-1* and *wah-1* genes (S1 Fig).

### Principal Component Analysis of the Variation in Gene Expression

**Fig 7:**
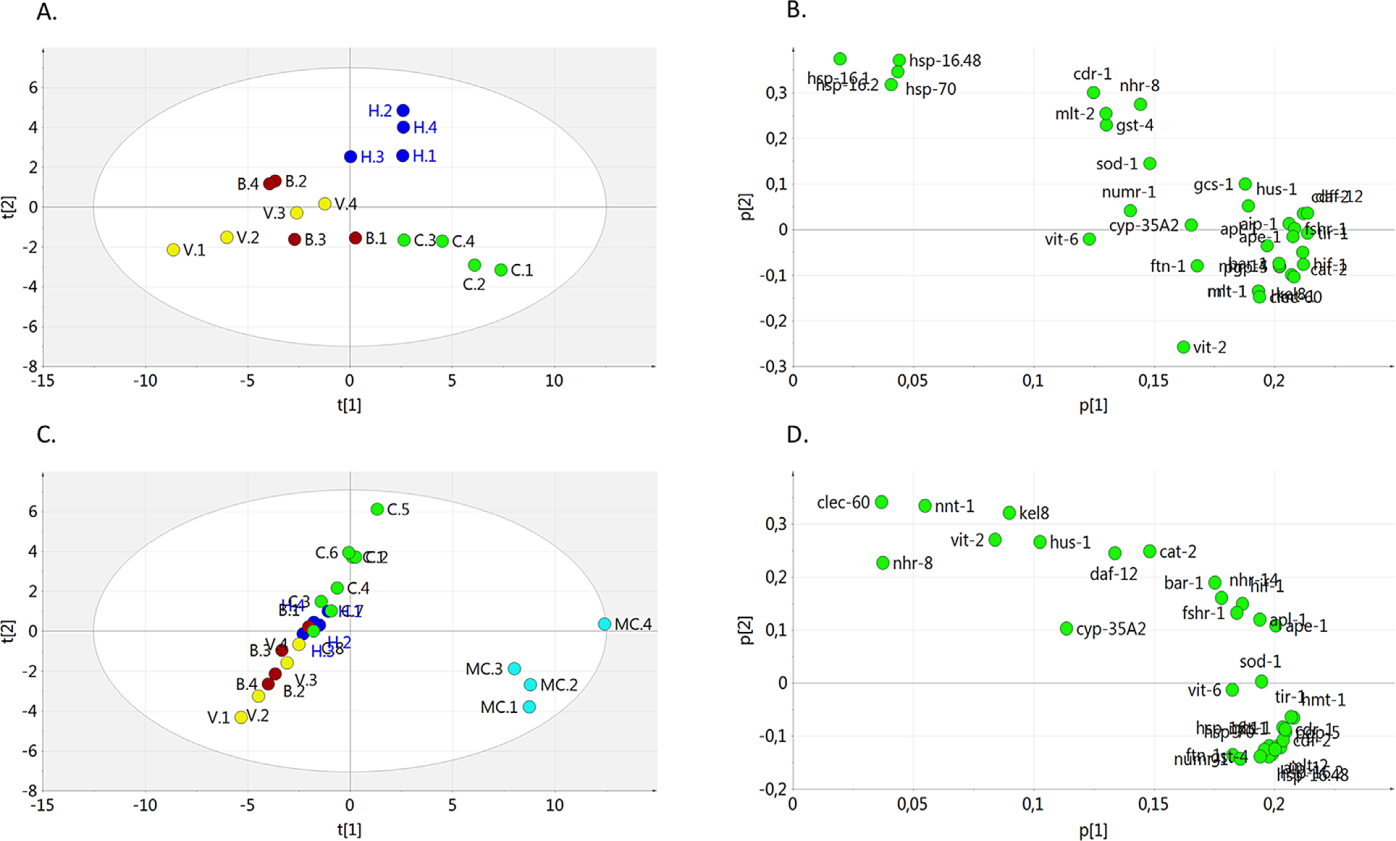
Principal component analysis. The PCA analysis was based on the gene expression pattern in *C*. *elegans* exposed to water samples from lake Hornträsket (H), Vormbäcken stream at Björkås (B), Vormbäcken stream at the outlet to Vindelälven (V), K-medium prepared with Milli-Q water (C) and metal cocktail (MC). A. Score plot showing the distribution following exposure to environmental waters and control water based on gene expression. The ellipse shows Hotellings T^2^ (0.05). B. PCA loading plot showing the distribution of the measured variables following exposure to the environmental waters and the K-medium control. C. Score plot showing the distribution following exposure to the MC, environmental waters and control water based on gene expression. The ellipse shows Hotellings T^2^ (0.05). D. PCA loading plot shows the distribution of the measured variables following exposure to the MC, environmental waters and the control (K-medium prepared with Milli-Q water).

In order to analyze the correlations between gene expression and metal exposures we performed PCA analysis of the data. A PCA score plot comparing sites Lake Hornträsket (H), Vormbäcken at Björkås (B) and at Vindelälven (V) and control (C, K-medium prepared with Milli-Q water) provided a model (R^2^X = 0.783, Q^2^ = 0.662, a two component model) showing that lake Hornträsket was distant from the other sites and the control (Fig 7A). Analysis of the score plot showed that principal component (PC) 1 and PC2 had Eigen-values of 9.56 and 2.97. The PC1 explained 59.7% of the variation, while PC2 explained 18.6% of the observed variation. Next, a loading plot was generated to show the distribution of the variables (Fig 7B). The data shows that the four heat-shock genes were positively correlated to lake Hornträsket (H) in the score plot (Fig 2 and Fig 7A). In addition, the gene expression differed between lake Hornträsket (H) and the two sites in Vormbäcken stream (B and V). While *pgp2* and *vit2* were down regulated in all three locations there were a further 13 genes down regulated at site B and 16 genes down regulated at site V (Figs 2–6 and Fig 7A). In order to compare the effects of water collected from the environment to the MC, we performed further PCA analysis. Including the MC which gave a model (R^2^X = 0.872, Q2 = 0.824, two component model) showing that the MC was distant from the other samples (Fig 7C). Analysis of the score plot showed that PC1 and PC2 had Eigen-values of 15.9 and 5.07. The PC1 explained 66.1% of the variation while PC2 explained 21.1% of the observed variation. A loading plot was generated to show the distribution of the variables (Fig 7D). The data showed that the upregulation of 18 genes (Figs 2–6) were positively correlated to the MC, confirming that there was a difference in gene expression between MC and the environmental waters. Taken together the PCA analysis confirmed that the water from lake Hornträsket (H) differed from waters collected from the Vormbäcken stream (sites B and V) and that all of the environmental waters show large differences in gene expression from the MC.

## Discussion

The mechanisms of toxicity of individual and simple mixtures of transition metals have been extensively studied in laboratory settings at the cellular and organism levels [25–27]. Surprisingly, the effects of complex aquatic environmental mixtures containing transition metals have rarely been evaluated. Naturally occurring concentrations of individual metals reproduced in the laboratory have elicited strong effects on physiology and gene expression [4,28]. Our study is one of the first systematic analysis of genetic response to metal mixtures clearly demonstrating that when the same metal concentrations are placed into their environmental context, the effects seen in the laboratory mixtures were abrogated. This indicates that there is a significant discrepancy in the biological effect elicited by samples collected in the field from laboratory reconstituted mixtures containing the same concentration of transition metals. Furthermore, the complexity of the mixtures in the aquatic environments contributed to a reduced or lack of effect on exposed organisms.

We established a gene expression profile of *C*. *elegans* responses to a simple mixture of Zn, Cu and Cd and compared it to a naturally occurring aquatic concentration gradient of the same metals in waters near a mining site in Sweden. The expression profiles of genes associated with stress response, metal toxicity, development, nuclear receptors, innate immunity and apoptosis resulted in a comprehensive view of the overall response. Studies have shown that changes in the transcriptome profile is more sensitive to environmental stress leading to physiological changes that correlate well with protein profiles [29, 30]. We therefore used changes in transcript levels to elucidate the stress response. The rationale for the selection of genes in this study was to include key responders and regulators of stress responses or environmental insult based on discrete studies reported in the literature. The laboratory prepared MC had two purposes: to validate the specificity of the selected genes and to determine if the responses to a simple laboratory prepared MC would also be exhibited in environmental water samples. The results of the MC exposure demonstrated that the effects observed in a simple metal mixture (MC) cannot be used to predict the biological effects of complex aquatic environments. Thus, while many biomarkers of exposure show good correlation to specific inducers, such as metals, the dose-response relationships cannot always be transferred to the more complex environmental situations. It was striking that the original environmental water samples exerted much lower, and sometimes opposite, effects on the *C*. *elegans* than would be predicted from the response observed with the MC. The difference in biological effects can be attributed to the presence of dissolved organic carbon (DOC), metal complexes and metal speciation in environmental waters, thus reducing bioavailability of the metals as compared to the laboratory metal mixture. In addition, the three environmental water samples also differed in gene expression profiles as seen following the PCA analysis (Fig 7A). While the most contaminated site, lake Hornträsket (H), did not upregulate any of the metal responsive genes, it differed in gene expression from the two down-stream locations in Vormbäcken. It is striking that none of the heat-shock genes upregulated at site H, were upregulated at sites B and V. The levels of calcium (Ca), silicon (Si), magnesium (Mg), sodium (Na) and potassium (K) increased down-stream of lake Hornttärsket and the tailings dam (Table 2). The increased level of these metals and the reduction in Cd, Cu and Zn levels likely to contributed to the altered gene expression observed in the present study.

Laboratory studies were the basis for the established biological effects of transition metal exposures and dose response relationships. It is now generally accepted that transition metals including Cu, Cd, Fe, Zn and Co produce reactive oxygen and free radical species that can interact with nucleic acids, induce lipid peroxidation and irreversibly modify proteins [15, 23]. Excessive oxidative damage to proteins eventually leads to misfolding and aggregation that contribute to aging and neurodegenerative disorders [31]. A key finding in this study was that the *C*. *elegans* metal (Fig 4) and oxidative stress (Fig 3) response to the laboratory prepared MC was not observed with the environmental waters. In contrast the general stress response (Fig 2), including the expression of heat shock proteins, were observed in *C*. *elegans* treated with both MC and Lake Hornträsket water, however the impact of the environmental waters was much lower. The heat shock response is a highly conserved and ancient machinery that restores cellular homeostasis after an assault has occurred and manifests itself as a general stress response. The heat shock response is considered an effective indicator for environmental monitoring and stress [32–34]. This is normally a non-specific first-tier response indicating that the organism is stressed and can be activated by fluctuations in a wide range of stressors originating from abiotic or biotic sources [35]. Heavy metals, among many other types of stresses are known to up regulate the heat shock protein genes [7, 36, 37]. In contrast, the metal response genes such as *mtl-1* and *mtl-2* that are highly specific to transition metal exposure were not up regulated in the aquatic samples. Multi-fold induction of the *mtl-1* and *mtl-2* genes by the MC correlates to previous reports on metal toxicity and knockout studies for these two genes indicating that the primers are functional [7, 38, 39]. The other metal responsive genes that were induced by MC, but not by the aquatic samples, including *ftn-1*, *pgp-5*, *cdf-2*, *htm-1*, *numr-1*, *aip-1* and *cdr-1*, have all previously been reported to be induced in the presence of heavy metals [36, 40–50].

Transition metals produce reactive oxygen species (ROS) and induce a strong oxidative stress response by up regulating the genes involved in detoxification, including *gcs-1*, *gst-4* and *sod-1* encoding for gamma-glutamine cysteine synthetase, glutathione-S-transferase and superoxide dismutase, respectively [7, 51]. This specific oxidative stress response was induced by the MC however, no effect on these genes was observed in *C*. *elegans* exposed to the aquatic samples. This indicates that ROS produced by transition metals or their effects are abrogated by the abiotic or biotic constitution of the environmental waters.

The chemical composition of environmental water samples containing complex mixtures of transition metals is routinely determined, however, little is known about the toxicity effects of these mixtures on living organisms. Different metal combinations in the laboratory have been shown to induce variable levels of toxicity on diverse organisms; either producing a neutralizing, additive or synergistic effects [36, 52, 53]. In a lethality study exposing *C*. *elegans* to 2 or 3 metals in the μM levels showed that after 48 h most transition metal combinations had either an additive or synergistic effect on the organism [36]. They showed that Cu combined with Zn or Cd produced a synergistic effect, while Zn was the only metal shown to neutralize other transition metal toxicity in *C*. *elegans* [36]. Notably, none of the metal concentrations in our study induced acute lethality in the exposed *C*. *elegans*. Xu et al [54] have corroborated the studies that binary mixtures often had synergistic effect, while studies on sea urchin embryos using ternary and quaternary metal mixtures showed that additive effects were predominant. This indicates that different organisms may have different responses to complex metal mixtures. Natural aquatic environments contain many abiotic and biotic substances that may interact with each other to affect bioavailability and in turn toxicity. In our aquatic samples, high levels (mg/l) of Ca, Si, Mg, Na and K, compared to other metals present in trace amounts (μg/l) were measured (Table 1). It is not known if the other metals contributed to neutralizing the biological response observed in the MC exposure and the differences between site H and sites B and V. The present results therefore suggest that chemical analysis on its own is not sufficient to characterize toxicological effects of aquatic environments and subsequent impact on living organisms.

The biotic ligand model (BLM) was developed and validated for individual metals (e.g. Cu, Zn, Ni) (e.g. see www.bio-met.net; www.pnec-pro.com). These models aim to predict local “predicted no-effect concentration” (PNEC) values taking the site specific physico-chemical water conditions into account, hence considering bioavailability in its toxicity estimations. Using the Bio-met tool and Lake Hornträsket site specific pH (6.7–6.8), DOC (4.8–5 mg/l) and Ca (6.2 mg/l), local EQS based PNEC values were calculated to approximately 16 μg/l for Cu and 34.5 μg/l for Zn. Compared to the measured concentration of Cu and Zn, this would indicate that Zn (bioavailability considerations as modeled by BLM included) should be the major contributor of toxicity. Corresponding BLM risk characterization ratio (RCR) was 11.8 for Zn and 1.8 for Cu indicating that a substantial risk is present from this metal exposure, and in particular from Zn. Measured concentrations of Cd, Zn and Cu compared to EQS [54, 55], (Cd, 0.08 μg/l class1) and Swedish general guideline limits (Cu, 4μg/l and Zn, 3 μg/l for waters with a hardness < 24 mg/l CaCO_3_, Naturvårdsverket, 2009) indicate high RCRs of 6.2, 7.3 and 136, respectively. Bioavailability estimates taken into consideration by means of the BLM largely lowers the RCRs for Cu and Zn and seems more closely related to the measured effects in the current study than do general guideline limit values.

The present study suggests that the masking effect of the specific matrix in Lake Hornträsket is even larger than predicted by BLM, and that very little effect is seen in the water from Lake Hornträsket, despite higher concentrations of metals. Although BLM is based on a large amount of ecotoxicological data, the vast majority of this data consists of controlled exposures based on growth media where metal salts and other constituents are added to simulate different exposure scenarios [56]. While it is considered good laboratory practice to perform such tests according to standard and reproducible methods, there will inevitably be differences compared to an environmental matrix. In most cases, bioavailability will be higher in laboratory settings due to speciation differences, thus, the results have a built-in conservative approach and will likely overestimate toxicity compared to field conditions. This may explain some of the differences seen in the present study when comparing measured effects and presumed effects obtained from guideline limits or from site specific modeling using the BLM.

The public concerns regarding cocktail and synergistic effects of pollutants in the environment have come under review by regulatory boards [57]. Synergistic effects, where normally toxic substances potentiate their toxicity in mixtures have been shown in the laboratory, but rarely in the environment at natural concentrations. Similarly the cocktail effect, where normally non-toxic compounds in combination produce toxic effects, has also been presented in lab experiments. A recent comprehensive review has reported that combinatory effects (synergistic, additive or cocktail effects) are rarely observed, as the particular chemical combinations at realistic concentrations are not often encountered naturally in the environment, occurring only in approximately 5% of the tested combinations [11]. Synergistic effects are also often found at high concentrations not normally found in the environment [11]. Our study using *C*. *elegans* and sensitive molecular techniques revealed that the synergistic biological effects seen in the laboratory mixtures of transition metals at natural concentration levels are either diminished or abrogated in complex aquatic environments. An intriguing insight from the present study is that although exposure to the MC caused alteration of expression in several genes those changes were not obvious in the environmental samples. Therefore, threshold values for the activation/inactivation of these biomarker genes cannot be extrapolated from laboratory studies but must be used in a site-specific context. The mechanisms and biomarkers used in the present study are common to other organisms as well, thus the results for *C*. *elegans* are relevant for assessing risks to other organisms. Consequently, extrapolation of laboratory toxicity studies to the environment based on chemical analysis and simple chemical mixtures are not indicative of the actual effects observed in the environment. In addition, as seen by the differences between sites H, B and V, no two environmental sites are the same, it is therefore vital that site-specific analyses for biological responses and toxicity are performed in order to obtain data for accurate risk assessment.

## Supporting Information

S1 Fig Genes that did not show significant change in expression in *C*. *elegans* exposed to the metal cocktail and environmental waters. Genes exhibiting no change in expression from the control in *C*. *elegans* treated with the metal cocktail (MC) or waters from Lake Hornträsket (H) and Vombäcken stream at Björnås (B) and the exit to Vindelälven (V). Genes include those associated with heat shock response (*hsf-1*, *sip-1*), oxidative stress response (*osr-1*, *skn-1*, *prdx-2*, *daf-2*), development (*fem-1*), innate immunity (*lys-7*, *abf-2*, *tol-1*) and apoptosis (*cep-1*, *wah-1*). One way analysis of variance (ANOVA) followed by Tukey’s post-test was used for multiple group comparison where * refers to *p*<0.01 as compared to control and # represents significant differences between the sites H, B and V. The dotted line represents the expression level normalized to that of the control (K-medium prepared with Milli-Q water).
(TIF)
