# Supplementary figures and images for: Comparative Analysis of Stress Induced Gene Expression in Caenorhabditis elegans following Exposure to Environmental and Lab Reconstituted Complex Metal Mixture
Source: PLoS One. 2015 Jul 13;10(7):e0132896. doi: 10.1371/journal.pone.0132896 (PMC4500601; doi:10.1371/journal.pone.0132896)

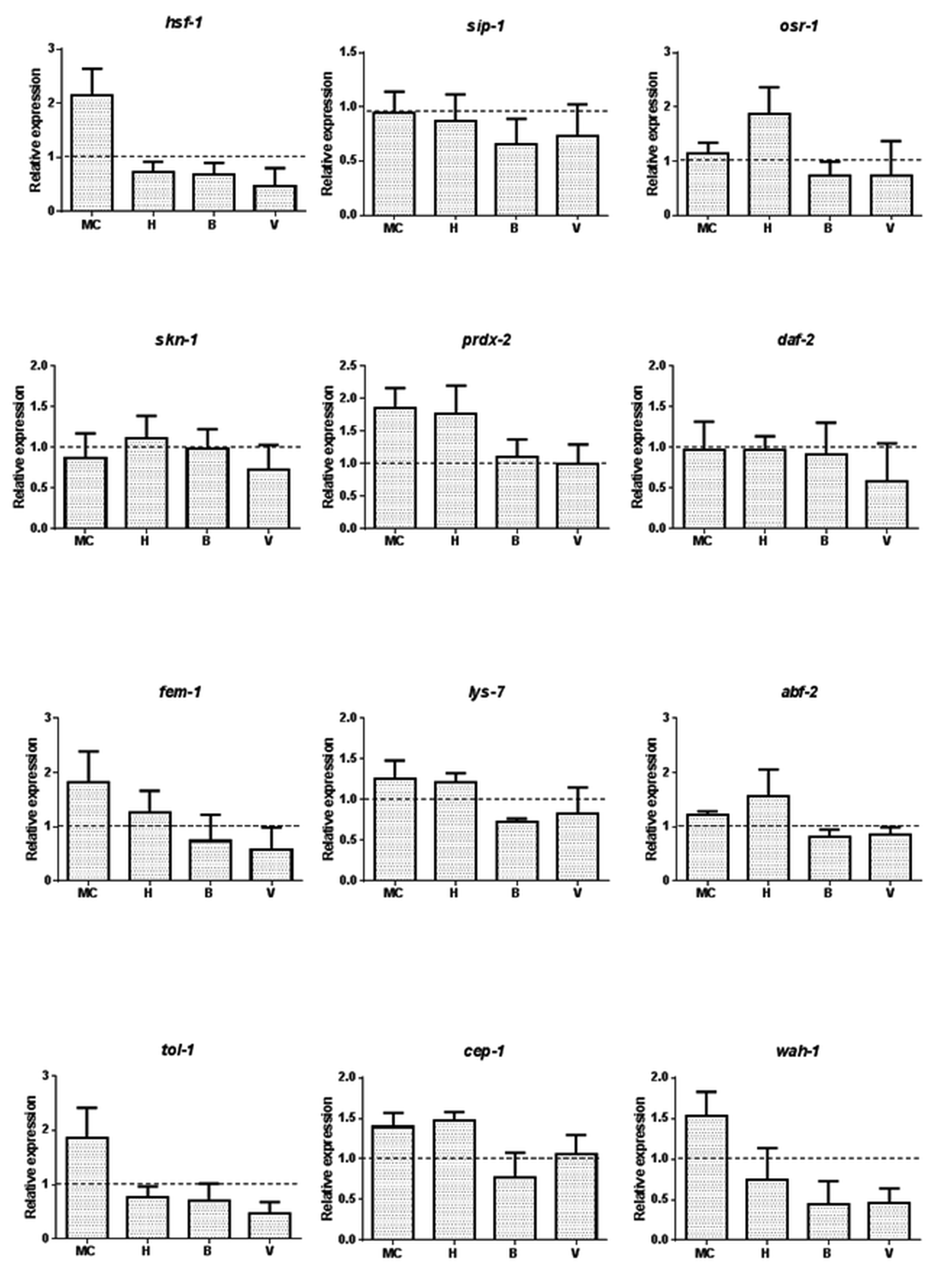

Supplement: S1 Fig — Genes exhibiting no change in expression from the control in C. elegans treated with the metal cocktail (MC) or waters from Lake Hornträsket (H) and Vombäcken stream at Björnås (B) and the exit to Vindelälven (V). Genes include those associated with heat shock response (hsf-1, sip-1), oxidative stress response (osr-1, skn-1, prdx-2, daf-2), development (fem-1), innate immunity (lys-7, abf-2, tol-1) and apoptosis (cep-1, wah-1). One way analysis of variance (ANOVA) followed by Tukey’s post-test was used for multiple group comparison where * refers to p<0.01 as compared to control and # represents significant differences between the sites H, B and V. The dotted line represents the expression level normalized to that of the control (K-medium prepared with Milli-Q water). (TIF) [file pone.0132896.s001.tif]
